# Bone Fragility in Turner Syndrome: Mechanisms and Prevention Strategies

**DOI:** 10.3389/fendo.2016.00034

**Published:** 2016-04-26

**Authors:** Maria Felicia Faienza, Annamaria Ventura, Silvia Colucci, Luciano Cavallo, Maria Grano, Giacomina Brunetti

**Affiliations:** ^1^Pediatrics Unit, Department of Biomedical Sciences and Human Oncology, University of Bari “A. Moro”, Bari, Italy; ^2^Section of Human Anatomy and Histology, Department of Basic Medical Sciences, Neuroscience and Sense Organs, University of Bari “A. Moro”, Bari, Italy; ^3^Department of Emergency and Organ Transplantation (DETO), University of Bari “A. Moro”, Bari, Italy

**Keywords:** Turner syndrome, bone, fractures, bone mineral density, estrogens

## Abstract

Bone fragility is recognized as one of the major comorbidities in Turner syndrome (TS). The mechanisms underlying bone impairment in affected patients are not clearly elucidated, but estrogen deficiency and X-chromosomal abnormalities represent important factors. Moreover, although many girls with TS undergo recombinant growth hormone therapy to treat short stature, the efficacy of this treatment on bone mineral density is controversial. The present review will focus on bone fragility in subjects with TS, providing an overview on the pathogenic mechanisms and some prevention strategies.

## Introduction

Turner syndrome (TS) is a congenital disease caused by partial or complete loss of one sex chromosome, which occurs in 1:2500 female live births. Monosomy 45,X0 is present in about 45% of cases (1), while the remaining TS patients show mosaic patterns with one or more additional cell lines and structural aberration (mostly Xq isochromosomes) (1).

The major phenotypic characteristics of TS subjects are short stature, ovarian dysgenesis, cardiac anomalies, and neurocognitive problems (2). Additionally, a low bone mineral density (BMD) and an increased risk of fractures have been described (3–5). The pathogenic mechanisms responsible of bone impairment in TS have not been completely elucidated. It is not clear whether the bone fragility is due to X-chromosomal abnormalities or is caused by estrogen deficiency that occurs during developmental age and in adulthood (3, 6, 7). Moreover, although many girls with TS undergo recombinant growth hormone (rGH) therapy to treat short stature in childhood, the efficacy of this treatment on BMD is controversial.

The present review will focus on bone fragility in subjects with TS, providing an overview on the underlying mechanisms and some options for management of bone impairment.

## Bone Fragility in TS

Low BMD and osteoporosis are clinical features in women with ovarian failure caused by TS, affecting these subjects often two to three decades earlier than that noted in postmenopausal osteoporosis (3, 8). According to a large epidemiological study, the risk of fractures for TS women seems to be about two times higher than general population, especially at metacarpal bones, femoral neck, lower spine, and forearm (9).

A fracture prevalence of 32.2%, and the forearm as the site most affected, has been reported by other authors (4). Two peaks of incidence have been observed, during childhood (non-osteoporotic fractures) and after 45 years (osteoporotic fractures) (4).

However, the fracture data in TS are controversial. In particular, the fracture prevalence may have been overestimated in studies on older patients, who had never been treated with estrogens or had received a delayed and suboptimal therapy (9).

Several studies have demonstrated that the detection of a low BMD in TS subjects by dual-energy X-ray absorptiometry (DXA) has some limits, as the influence of body size on areal BMD (aBMD) interpretation (10). Smaller bones project less density on the measured surface than bigger ones, resulting in lower *T*- and *Z*-scores in short individuals (11). In fact, the apparent BMD deficit in TS subjects is reduced when the reduced size of bones has been taken into account (3, 11).

The development of new bone imaging diagnostics, as peripheral quantitative computed tomography (pQCT), has allowed the evaluation of bone geometry and bone compartmental characteristics of TS subjects. In fact, pQCT provides precise measurements of three-dimensional bone density without the influence of bone size, and leads to assess trabecular and cortical bone density independently (12).

In this respect, using pQCT, a low cortical BMD, with marked thinning of the bone cortex, and normal trabecular BMD of radial bone have been observed in TS adolescents (12), and similar results were found in TS young adults (13). These findings suggested that selective decrease of cortical bone may confer biomechanical disadvantage and predispose TS subjects to bone fragility and fractures (12).

Nevertheless, other studies have documented a reduction of both cortical and trabecular bone in TS subjects during and after puberty by using pQCT (14), whereas a normal (15) or increased cortical BMD (16) has been observed by using high-resolution pQCT (HR-pQCT). Table 1 collects the principal studies on BMD measurements in TS subjects.

**Table 1:** **BMD measurements in TS patients**.

Population Sample (*n*)	Age (years)	BMD measurement	Outcome	Reference
40 TS patients	16–45	DXA at the lumbar spine and femoral neck	Subjects with TS and those with primary amenorrhea had severe osteopenia compared with healthy controls	Davies et al. (8)
40 subjects with primary amenorrhea				
40 healthy controls				
9 TS patients with spontaneous puberty	14.9 ± 1.3	DXA at the lumbar spine	BMD values were in the normal range in TS patients with spontaneous puberty and in the osteopenia range in puberty induced group.	Carrascosa et al. (17)
18 puberty induced TS patients (adolescent)	16.8 ± 0.7			
10 puberty induced TS patients (young adult)	20.9 ± 0.7			
21 GH and estrogen treated TS patients	19.5 ± 2.3	pQCT at the distal metaphysis and at the proximal diaphysis	BMC was decreased, resulting in a low total vBMD. This was due to decreased cortical thickness at both sites of measurement, whereas trabecular vBMD of the metaphysis was normal	Bechtold et al. (13)
40 TS patients	34.0 ± 11.0	DXA at the lumbar spine and femoral neck	Mean areal bone density was significantly lower at the lumbar spine and femoral neck in TS women than in controls	Bakalov et al. (11)
43 controls	32.0 ± 8.0			
23 GH-treated TS patients	21.5 ± 9.4	DXA at RAD-1/3, RAD-UD, AP spine L1–L4, total hip	There was no significant difference in the two groups in BMD or BMAD at the wrist, hip, spine, or whole-body BMC	Bakalov et al. (18)
23 non-GH-treated TS patients	21.7 ± 9.4			
68 TS patients belonging to three age groups with different rGH treatment	6.1 ± 2.1	Phalangeal radiographic absorptiometry	Most untreated young TS patients have a normal vBMD. During 7 years of GH treatment the BMD SD score increased significantly	Sas et al. (19)
	6.7 ± 2.4			
	6.5 ± 2.4			
60 TS patients	36.7 ± 9.6	DXA at the lumbar spine (L2–L4), the hip (femoral neck and trochanteric region), and the non-dominant forearm	BMC and aBMD were universally reduced in TS, whereas vBMD was slightly reduced in the spine	Granvholt et al. (3)
59 controls	36.0 ± 9.5			
50 TS patients	30–59	DXA at the lumbar spine (L2–L4); QCT at vertebral bodies L1–L2	Lumbar spine BMD was significantly reduced in women not taking ERT according to current guidelines	Hanton et al. (20)
21 TS patients	20–40	DXA at the lumbar spine and proximal femur	The bone mineral density at the lumbar spine and proximal femur increased after 3 years of HRT	Khastgir et al. (21)
16 TS patients	29.1 ± 5.2	DXA at the lumbar spine (L1–L4) and of whole body	BMD of lumbar spine and whole body of TS were significantly lower than age-matched normal subjects	Suganuma et al. (22)
83 TS patients	12.76 ± 4.4	TB, LS, and FN DXA	The results show a height-independent prepubertal decrease in bone mass accrual, which ceased with puberty	Högler et al. (23)
22 TS patients	12.7 ± 3.8	DXA at the lumbar spine and femur and pQCT scanning of the non-dominant forearm, distal metaphyseal site, and the proximal diaphysis of radius	TS is associated with reduced BMAD at the femoral neck; pQCT data suggest that cortical density is reduced with sparing of trabecular bone	Holroyd et al. (12)
21 controls	12.9 ± 3.8			
67 TS patients	6.0–19.4	pQCT at the non-dominant radius	Cortical vBMD was decreased in all TS patients. Height-, age-, and cortical thickness-adjusted cortical vBMD were positively correlated with the duration of GH therapy and to estrogen administration. Girls with a history of fractures had lower total vBMD at the metaphysis compared to non-fractured TS girls	Soucek et al. (14)
32 TS patients	35 (20–61)	HR-pQCT at non-dominant distal radius and tibia	TS patients had compromised trabecular microarchitecture and lower bone strength at radius and tibia	Hansen et al. (16)
32 controls	36 (19–58)			
22 TS patients	10.3 ± 2.2	pQCT at forearm	Trabecular BMD and bone strength index were normal in TS as well as SHOX-D patients. Increased total bone area and thin cortex were observed at the proximal radius for TS and SHOX-D patients	Soucek et al. (24)
10 children with SHOX-D	10.3 ± 2.1			
24 adolescent TS patients	17.1 ± 3.1	DXA at the lumbar spine and femoral neck; phalangeal QUS	Adolescents with TS had a normal lumbar vBMD and a reduced vBMD at the femoral neck. Phalangeal QUS represents a useful method to identify subjects with increased fracture risk	Vierucci et al. (25)
60 TS patients belonging to three age groups	5.94 ± 3.27	DXA at the lumbar spine (L2–L4)	Bone impairment was detectable by DXA in subjects aged under 10, although it became more evident with aging	Faienza et al. (26)
	13.51 ± 2.06			
	23.45 ± 6.80			
88 TS patients with primary amenorrhea	17–58	DXA at the lumbar spine (L2–L4)	TS patients receiving late initiation of HRT had a BMD significantly lower than the early initiation group or spontaneous menstrual cycles group	Nakamura et al. (27)
12 TS patients with spontaneous menstrual cycles	18–40			
32 TS patients	15.3 ± 3.2	pQCT at the non-dominant radius and tibia	Whereas the cortical BMD was decreased in the radius, it was increased in the tibia. After correcting the cortical BMD for the partial volume effect, the mean *Z*-score was normal in the radius in TS. The corrected cortical BMD values were similar in the radius and tibia	Soucek et al. (15)
28 TS patients	17–45	DXA of lumbar spine, hip, and radius and HR-pQCT scans of the radius and tibia	No significant difference in DXA-derived BMD and HR-pQCT micro-architectural parameters was detected between childhood GH treatment compared to no treatment in TS	Nour et al. (10)

*AP, anteroposterior; BMC, bone mineral content; BMD, bone mineral density; aBMD, area BMD; vBMD, volumetric BMD; BMAD, bone mineral apparent density; DXA, dual-energy X-ray absorptiometry; TS, Turner syndrome; FN, femoral neck; GH, growth hormone; rGH, recombinant GH; HRT, hormone replacement therapy; LS, lumbar spine; pQCT, peripheral quantitative computed tomography; HR-pQCT, high-resolution pQCT; QUS, quantitative ultrasound; RAD-1/3, one-third proximal radius; RAD-UD, ultradistal radius; TB, total body*.

These conflicting findings are due to different diagnostic imaging and characteristics of TS subjects (10). In particular, HR-pQCT provides a refined image resolution, which permits detailed evaluation of bone microarchitecture, eliminating the partial volume artifacts that may influence cortical BMD measurements, determined with pQCT (15, 16). Moreover, studies which assessed BMD in adolescent TS patients may not be appropriately matched with healthy controls (12). In fact, most TS individuals under 13 years of age do not have completed puberty, whereas the majority of age-matched healthy girls could have achieved menarche with a significant increase in bone mass accrual (28).

Therefore, different factors rather than reduced cortical BMD might predispose TS subjects to higher risk of fractures, such as altered bone geometry (bone size, shape, and microarchitecture) and decrease of trabecular BMD or motor dysfunction (29).

The bone microarchitecture is influenced by the continuous remodeling, a coordinated process between formation and degradation of bone, respectively, managed by osteoblasts (OBs) and osteoclasts (OCs). The by-products of these processes can be quantified in serum and urine, and used as markers of the balance in bone activity (30, 31). Markers of bone formation are represented by alkaline phosphatase (ALP), bone-specific ALP, procollagen I-amino-terminal propeptide (PINP), procollagen III-amino terminal propeptide (PIIINP), and osteocalcin, while markers of bone resorption comprise C-telopeptide fragments of type 1 collagen (ICTP), carboxy-terminal telopeptide of type 1 collagen (CTX), and N-terminal cross-linking telopeptide of type 1 collagen (NTX). In TS, analysis of these biochemical markers revealed an increased resorption of bone, with normal or decreased bone formation, suggesting imbalance in bone remodeling (3, 26, 32). However, little is known about the relation of aBMD and volumetric BMD (vBMD) to bone markers in young TS (8, 33, 34).

Furthermore, as skeletal muscle contraction represents a mechanical stimulus for bone development (35–37), it was suggest that impaired motor skills and reduced muscle power described in TS, may contribute to increased fracture rate (29).

## Pathogenetic Mechanisms of Bone Fragility in TS

The mechanisms responsible of bone fragility in TS have not been completely elucidated. While some authors ascribed bone fragility to estrogen deficiency occurring during development and in adulthood (17, 20, 38), other researches sustain a direct or indirect effect of X-chromosomal abnormalities (14, 39, 40). Moreover, some comorbidities of the syndrome could be involved in bone loss. Finally, the effect of rGH therapy on BMD remains to be clarified.

**Figure 1:**
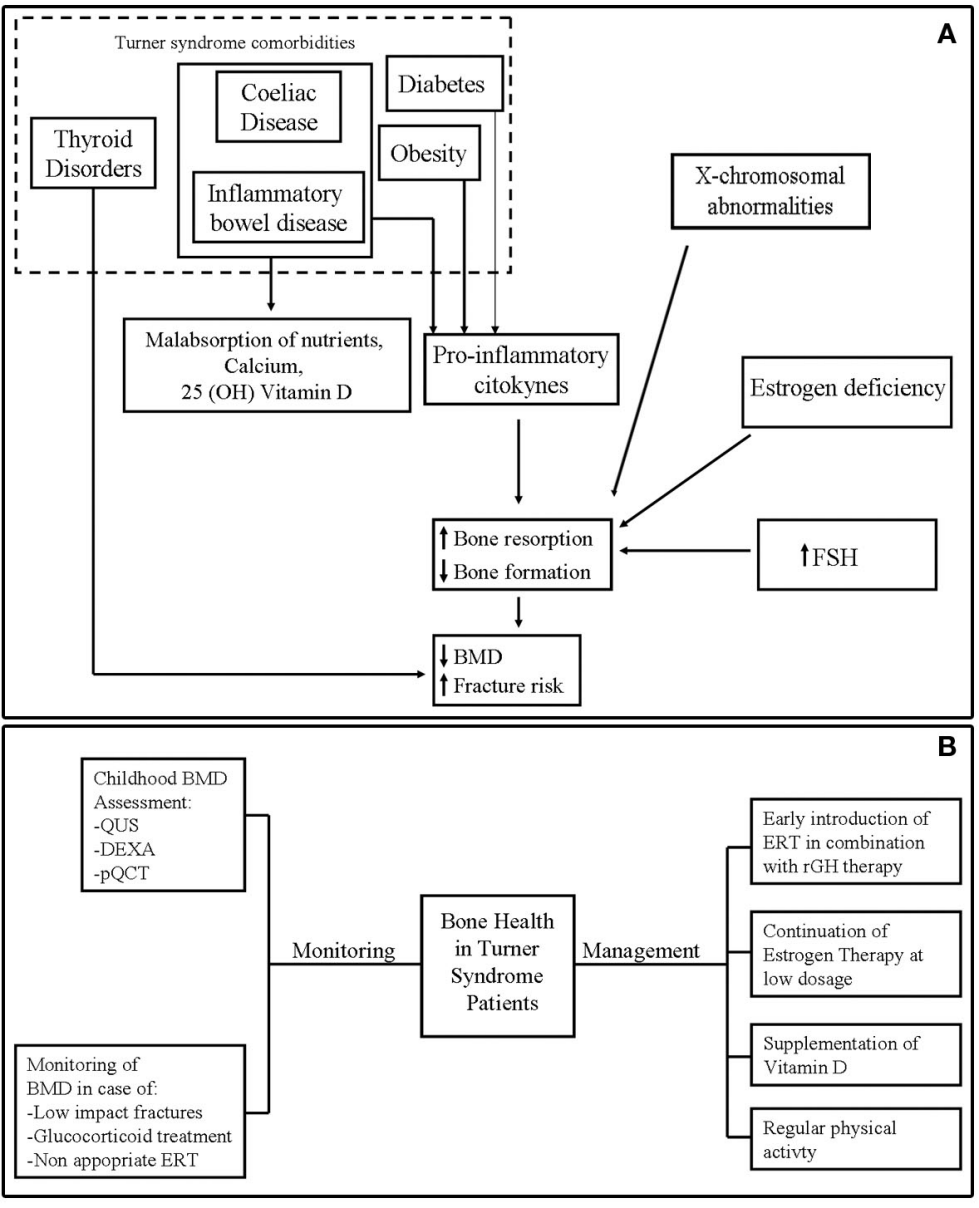
**(A)** Potential mechanisms of bone fragility in Turner syndrome. **(B)** Monitoring and management of bone health in Turner syndrome.

Figure 1A shows the potential mechanisms of poor bone health in TS.

### Estrogen Deficiency

Ovarian dysfunction is a common complication in TS subjects, and estrogen replacement therapy (ERT) is needed for the induction of puberty and for the maintenance of bone and cardiac health (2).

The effects of estrogens on bone seem to be dose-dependent: low levels of exposure may increase the mechanical sensitivity of the periosteum, whereas higher concentrations may inhibit periosteal apposition, reducing cortical thickness (41). Although the appropriate estrogen dosage to treat hypogonadism and the timing of the therapy are still debated (27), estrogen replacement has been shown to be important to avoid a rapid decrease in BMD and to optimize bone mineral accretion in TS adolescents (23). In particular, early initiation of ERT is more effective on bone mass acquisition (27), and low-dose administration in childhood has also neurocognitive and behavioral benefits (42). The possible downside of early ERT is the reduction of adult height by accelerating epiphyseal fusion, which can be avoided by combining ultra-low-dose estrogen with rGH therapy.

An increased BMD due to augmented trabecular bone volume, and an unchanged cortical bone, has been demonstrated after 3 years of treatment with subcutaneous estradiol implants in TS young women (21). These findings suggested a role of estrogens not only in preventing bone loss but also in increase of bone mass.

In adult TS patients, the assumption of exogenous estrogen is important for prevention of osteoporosis (43) and in reducing risk factors for atherosclerosis (44, 45), but the optimal dose at the different ages, administration route, type of estrogen and gestagen remain to be determined (46).

### Follicle-Stimulating Hormone

Follicle-stimulating hormone (FSH) has a well-established role in reproduction, stimulating ovarian folliculogenesis, and estrogen synthesis, but recently has emerged also a role in bone metabolism. Some studies in mouse or human cells showed a positive direct or indirect FSH effect on OC differentiation and function (47).

In particular, FSH enhances osteoclastogenesis and bone resorption directly by binding to FSH receptor expressed on OCs and their precursors (47), and indirectly by triggering the production of tumor necrosis factor-α (TNF-α) from bone marrow macrophages, granulocytes, and T-cells (48). This proinflammatory cytokine stimulates receptor activator of nuclear factor kappa-B ligand (RANKL) expression and synergizes with RANKL signaling to maximize OC formation (49).

High FSH circulating levels have been associated with bone loss (50) and with bone turnover markers in pre- and perimenopausal women (51). Furthermore, hypergonadotropic amenorrheic women have low BMD because of a potential direct effect of FSH on bone metabolism (52), while decrease in FSH serum levels resulting from estrogen therapy is associated with raise in bone mass (53).

Girls with TS have a biphasic pattern of gonadotrophin levels with high FSH and LH serum levels during childhood and at the time of expected puberty, in subjects with monosomy 45,X0 respect to those with mosaicism (54). A recent study by Faienza et al. demonstrated a high osteoclastogenic potential in girls and young women with TS, before and after puberty induction. This osteoclastogenic potential seems to be supported by the elevated FSH serum levels at prepubertal stage, before ERT, and by high RANKL levels in young women on continuous ERT (26). These results confirmed the indirect action of FSH in stimulation of osteoclastogenesis, through increase of TNF-α by bone marrow macrophages/granulocytes and T-cells (55) and by promoting the expression of receptor activator of nuclear factor kappa-B (RANK) on human monocytes (56).

### X-Chromosomal Abnormalities

Haploinsufficiency of the X-linked genes (57, 58) that escapes X inactivation (59) is one of the key factors responsible for clinical phenotype of TS. In particular, haploinsufficiency of *SHOX* gene, located in the pseudoautosomal region of sexual chromosomes, seems to play a key role in growth failure typical of these subjects (27, 57). Furthermore, SHOX deficiency (SHOX-D) has been proposed as the most probable gene deficit responsible for the other skeletal alterations in TS, such as short metacarpals, high-arched palate, cubitus valgus, Madelung deformity, and mesomelia (58, 60). Moreover, despite the exact molecular-genetic effect of *SHOX* on bone is not clear, and limited informations are available on the intracellular pathways activated by *SHOX* (61, 62), it is suggested that isolated SHOX-D may alter bone geometry and microarchitecture, rather than bone strength (63). Similar changes in bone geometry at the proximal radius (increased total bone area and thin cortex) have been found in prepubertal TS girls and SHOX-D patients, suggesting that *SHOX* haploinsufficiency is the causative factor leading to the changes in shape and geometry of the radius observed in TS. Interestingly, altered bone geometry parameters were more pronounced in patients with isolated SHOX-D respect to TS, and this can be explained partially by preserved *SHOX* function in mosaic TS subjects (24).

Others genes potentially involved in bone abnormalities of TS have recently been identified through analysis of the transcriptome profiles of human 45,X0 and 46,XX fibroblast cells (64). In 45,X0 karyotype, the analysis revealed a downregulation of different genes, directly or indirectly associated with bone metabolism, such as bone morphogenetic protein 2 (*BMP2*), associated with bone mineralization; insulin-like growth factor 2 (*IGF2*), placental growth factor (*PGF*), and prostaglandin endoperoxide synthase 1 (*PTGS1*) involved in bone repair, formation, and development (65–70), and secreted frizzled-related protein 1 (*SFRP1*) associated with Wnt signaling and affecting OB proliferation and differentiation (71, 72). Further study on tissue-specific gene expression profiling will help to understand the molecular mechanism involved in bone abnormality of TS subjects.

### Effect of rGH Therapy on Bone Health in TS

Recombinant growth hormone therapy is widely used for treatment of growth failure in girls with TS, although TS patients are not GH deficient (GHD) (5).

The efficacy of rhGH therapy on BMD in TS is controversial (18, 73) also due to the small or lacking untreated control groups (12). Some studies suggest an improvement of bone density (19, 73) and some reported none effects (17), while others found decrease of BMD (18, 22).

A recent study evaluated the effects of GH treatment on bone in 28 young adults with TS using HR-pQCT. The bone strength was evaluated through measurements of finite element (FE) analysis and polar moment of inertia (pMOI) (10). The authors reported an increase in total bone size (length and cross-sectional area) and pMOI in GH-treated TS patients, while no significant differences in DXA-derived BMD, HR-pQCT microarchitectural parameter, and FE-estimated bone strength were found between treated and non-treated groups (10). These findings suggested that the higher pMOI and increase of bone size may reduce fracture risk in GH-treated TS subjects.

### Comorbidities Affecting Bone Health in TS

Different comorbidities of TS may affect bone health, such as obesity, diabetes, and some autoimmune disorders [celiac disease (CD), inflammatory bowel disease (IBD), and thyroid disorders].

Body composition is altered in TS with increased total and visceral fat mass, reduced lean mass, and augmented BMI (74, 75). Moreover, the risk of both type 1 and type 2 diabetes mellitus is increased (9), with fasting hyperinsulinemia and impaired glucose tolerance in 25–78% of adult TS patients (44, 76). Obesity, and in particular visceral adiposity, has been related to low BMD and greater fracture risk (77). Several evidences support this thesis. In particular, a strong relationship between inhibition of the OB formation and induction of the adipocyte differentiation has been demonstrated (78, 79). Moreover, the increased circulating and tissue proinflammatory cytokines in obesity may promote OC activity and bone resorption (80, 81). Diabetes is considered an important risk factor for fractures, even if the mechanisms responsible for greater bone fragility in diabetic patients remain to be elucidated (82, 83).

Celiac disease and IBD represent other clinical conditions affecting TS patients, which may predispose them to bone fragility (28), due to malabsorption of calcium and other macro- and microelements essential for bone metabolism, and the presence of chronic inflammation (84).

Hypothyroidism affects up to 70% of TS patients, often with autoimmune cause (85), and it is related to the increased risk of fractures, although the mechanism remains unclear (86).

## Management of Bone Fragility in TS

Strategies to prevent osteoporosis and fractures should been considered in TS subjects, just during pediatric age (87). In the first instance, bone mineral status should been assessed in childhood to detect TS subjects at increased risk of bone impairment and in order to carry out preventive measures (25). In particular, it is preferable to use diagnostic methods that take into account for reduced bone size of these patients, such as pQCT or volumetric transformation of DXA data (88).

Moreover, the use of non-invasive and radiation-free tools, such as phalangeal quantitative ultrasound (QUS), can be particularly useful for routinely assessing of BMD in children and adolescents with TS (25).

Bone mineral density evaluation should be repeated frequently in TS patients who were most exposed to bone fragility, such as subjects undergone to glucocorticoid treatment, with experience of low-impact fracture or in absence of adherence to ERT (5) (Figure 1B).

The main measures to optimize bone health in TS are represented by the timely introduction of ERT and continuation of estrogen therapy through young adult life according to consensus guidelines (26, 27, 89) (Figure 1B).

Many studies have found low serum vitamin D levels in TS subjects, which may contribute to the reduction of BMD (3, 33). Moreover, a positive correlation between bone size and density in TS, and level of physical activity has been reported (90). Thus, supplementation of vitamin D and active lifestyle, including weight bearing and regular physical activity, could confer benefit in maintaining bone health in TS (91).

## Conclusion

Although data on impairment of bone density and geometry and fracture risk are often controversial, bone fragility is recognized as one of the major lifelong comorbidities in TS subjects. The pathogenetic mechanisms responsible of bone impairment remain to be well clarified, although estrogen deficiency and X chromosomal abnormalities represent important factors. An enhanced spontaneous osteoclastogenesis has been demonstrated to occur in girls and young women with TS before and after pubertal induction with ERT. This process seems to be more active in girls before puberty induction and supported by the high FSH serum levels observed at prepubertal stage, while in young women on continuous ERT, the effects on OCs seem to be mediated mostly by high RANKL levels. We recommended to consider the average age of 9 years as a crucial time point for the introduction of ERT. In the future, a neutralizing FSH antibody could be useful. In the meantime, a regimen combining the earlier introduction of ERT with rGH treatment in girls with TS could have the effect to reduce FSH levels and to preserve bone health in these subjects.

## Author Contributions

All the authors wrote a section of the review and critically revised the manuscript.

## Conflict of Interest Statement

The authors declare that the research was conducted in the absence of any commercial or financial relationships that could be construed as a potential conflict of interest.

## References

[1] Wolff DJ Van Dyke DL Powell CM Working Group of the ACMG Laboratory Quality Assurance Committee. Laboratory guideline for Turner syndrome. Genet Med (2010) 12:52–5. 10.1097/GIM.0b013e3181c684b2 20081420

[2] Bondy CA Turner Syndrome Study Group. Care of girls and women with Turner syndrome: a guideline of the Turner syndrome study group. J Clin Endocrinol Metab (2007) 92:10–25. 10.1210/jc.2006-1374 17047017

[3] Gravholt CH Lauridsen AL Brixen K Mosekilde L Heickendorff L Christiansen JS. Marked disproportionality in bone size and mineral, and distinct abnormalities in bone markers and calcitropic hormones in adult Turner syndrome: a cross-sectional study. J Clin Endocrinol Metab (2002) 87:2798–808. 10.1210/jcem.87.6.8598 12050253

[4] Han TS Cadge B Conway GS. Hearing impairment and low bone mineral density increase the risk of bone fractures in women with Turner’s syndrome. Clin Endocrinol (2006) 65:643–7. 10.1111/j.1365-2265.2006.02643.x 17054467

[5] Bakalov VK Bondy CA. Fracture risk and bone mineral density in Turner syndrome. Rev Endocr Metab Disord (2008) 9:145–51. 10.1007/s11154-008-9076-2 18415020

[6] Ross JL Scott C Jr Marttila P Kowal K Nass A Papenhausen P Phenotypes associated with SHOX deficiency. J Clin Endocrinol Metab (2001) 86:5674–80. 10.1210/jcem.86.12.8125 11739418

[7] Nissen N Gravholt CH Abrahamsen B Hauge EM Jensen JE Mosekilde L Disproportional geometry of the proximal femur in patients with Turner syndrome: a cross-sectional study. Clin Endocrinol (2007) 67:897–903. 10.1111/j.1365-2265.2007.02984.x 17681028

[8] Davies MC Gulekli B Jacobs HS. Osteoporosis in Turner’s syndrome and other forms of primary amenorrhoea. Clin Endocrinol (1995) 43:741–6. 10.1111/j.1365-2265.1995.tb00544.x 8736278

[9] Gravholt CH Juul S Naeraa RW Hansen J. Morbidity in Turner syndrome. J Clin Epidemiol (1998) 51:147–58. 10.1016/S0895-4356(97)00237-0 9474075

[10] Nour MA Burt LA Perry RJ Stephure DK Hanley DA Boyd SK. Impact of growth hormone on adult bone quality in Turner syndrome: a HR-pQCT study. Calcif Tissue Int (2016) 98:49–59. 10.1007/s00223-015-0064-8 26439721

[11] Bakalov VK Chen ML Baron J Hanton LB Reynolds JC Stratakis CA Bone mineral density and fractures in Turner syndrome. Am J Med (2003) 115:259–64. 10.1016/S0002-9343(03)00364-4 12967689

[12] Holroyd CR Davies JH Taylor P Jameson K Rivett C Cooper C Reduced cortical bone density with normal trabecular bone density in girls with Turner syndrome. Osteoporos Int (2010) 21:2093–9. 10.1007/s00198-010-1170-0 20135092

[13] Bechtold S Rauch F Noelle V Donhauser S Neu CM Schoenau E Musculoskeletal analyses of the forearm in young women with Turner syndrome: a study using peripheral quantitative computed tomography. J Clin Endocrinol Metab (2001) 86:5819–23. 10.1210/jcem.86.12.8063 11739445

[14] Soucek O Lebl J Snajderova M Kolouskova S Rocek M Hlavka Z Bone geometry and volumetric bone mineral density in girls with Turner syndrome of different pubertal stages. Clin Endocrinol (2011) 74:445–52. 10.1111/j.1365-2265.2010.03955.x 21138463

[15] Soucek O Schönau E Lebl J Sumnik Z. Artificially low cortical bone mineral density in Turner syndrome is due to the partial volume effect. Osteoporos Int (2015) 26:1213–8. 10.1007/s00198-014-2901-4 25288443

[16] Hansen S Brixen K Gravholt CH. Compromised trabecular microarchitecture and lower finite element estimates of radius and tibia bone strength in adults with turner syndrome: a cross-sectional study using high-resolution-pQCT. J Bone Miner Res (2012) 27:1794–803. 10.1002/jbmr.1624 22492464

[17] Carrascosa A Gussinyé M Terradas P Yeste D Audí L Vicens-Calvet E. Spontaneous, but not induced, puberty permits adequate bone mass acquisition in adolescent Turner syndrome patients. J Bone Miner Res (2000) 15:2005–10. 10.1359/jbmr.2000.15.10.2005 11028454

[18] Bakalov VK Van PL Baron J Reynolds JC Bondy CA. Growth hormone therapy and bone mineral density in Turner syndrome. J Clin Endocrinol Metab (2004) 89:4886–9. 10.1210/jc.2004-0481 15472180

[19] Sas TC de Muinck Keizer-Schrama SM Stijnen T van Teunenbroek A van Leeuwen WJ Asarfi A Bone mineral density assessed by phalangeal radiographic absorptiometry before and during long-term growth hormone treatment in girls with Turner’s syndrome participating in a randomized dose-response study. Pediatr Res (2001) 50:417–22. 10.1203/00006450-200109000-00019 11518831

[20] Hanton L Axelrod L Bakalov V Bondy CA. The importance of estrogen replacement in young women with Turner syndrome. J Womens Health (Larchmt) (2003) 12:971–7. 10.1089/154099903322643893 14709185

[21] Khastgir G Studd JW Fox SW Jones J Alaghband-Zadeh J Chow JW. A longitudinal study of the effect of subcutaneous estrogen replacement on bone in young women with Turner’s syndrome. J Bone Miner Res (2003) 18:925–32. 10.1359/jbmr.2003.18.5.925 12733734

[22] Suganuma N Furuhashi M Hirooka T Moriwaki T Hasegawa Y Mori O Bone mineral density in adult patients with Turner’s syndrome: analyses of the effectiveness of GH and ovarian steroid hormone replacement therapies. Endocr J (2003) 50:263–9. 10.1507/endocrj.50.263 12940454

[23] Högler W Briody J Moore B Garnett S Lu PW Cowell CT. Importance of estrogen on bone health in Turner syndrome: a cross-sectional and longitudinal study using dual-energy X-ray absorptiometry. J Clin Endocrinol Metab (2004) 89:193–9. 10.1210/jc.2003-030799 14715849

[24] Soucek O Zapletalova J Zemkova D Snajderova M Novotna D Hirschfeldova K Prepubertal girls with Turner syndrome and children with isolated SHOX deficiency have similar bone geometry at the radius. J Clin Endocrinol Metab (2013) 98:E1241–7. 10.1210/jc.2013-1113 23666967

[25] Vierucci F Del Pistoia M Erba P Federico G Saggese G. Usefulness of phalangeal quantitative ultrasound in identifying reduced bone mineral status and increased fracture risk in adolescents with Turner syndrome. Hormones (Athens) (2014) 13:353–60. 10.14310/horm.2002.1485 25079459

[26] Faienza MF Brunetti G Ventura A Piacente L Messina MF De Luca F Mechanisms of enhanced osteoclastogenesis in girls and young women with Turner’s syndrome. Bone (2015) 81:228–36. 10.1016/j.bone.2015.07.021 26208797

[27] Nakamura T Tsuburai T Tokinaga A Nakajima I Kitayama R Imai Y Efficacy of estrogen replacement therapy (ERT) on uterine growth and acquisition of bone mass in patients with Turner syndrome. Endocr J (2015) 62:965–70. 10.1507/endocrj.EJ15-0172 26289838

[28] Lucaccioni L Wong SC Smyth A Lyall H Dominiczak A Ahmed SF Turner syndrome – issues to consider for transition to adulthood. Br Med Bull (2015) 113:45–58. 10.1093/bmb/ldu038 25533182

[29] Soucek O Lebl J Matyskova J Snajderova M Kolouskova S Pruhova S Muscle function in Turner syndrome: normal force but decreased power. Clin Endocrinol (2015) 82:248–53. 10.1111/cen.12518 24890376

[30] Schoenau E Saggese G Peter F Baroncelli GI Shaw NJ Crabtree NJ From bone biology to bone analysis. Horm Res (2004) 61:257–69. 10.1159/000076635 14963367

[31] Szulc P Seeman E Delmas PD. Biochemical measurements of bone turnover in children and adolescents. Osteoporos Int (2000) 11:281–94. 10.1007/s001980070116 10928217

[32] Cleemann L Holm K Kobbernagel H Skouby SO Kristensen B Smedegaard H Normal tempo of bone formation in Turner syndrome despite signs of accelerated bone resorption. Horm Res Paediatr (2011) 76:193–201. 10.1159/000329046 21791892

[33] Landin-Wilhelmsen K Bryman I Windh M Wilhelmsen L. Osteoporosis and fractures in Turner syndrome-importance of growth promoting and oestrogen therapy. Clin Endocrinol (1999) 51:497–502. 10.1046/j.1365-2265.1999.00841.x 10583318

[34] Gallicchio CT Figueiredo-Alves ST Prendin TR Mendonca LM Farias ML Guimaraes MM Effect of puberty on the relationship between bone markers of turnover and bone mineral density in Turner’s syndrome. Horm Res (2004) 61:193–9. 10.1159/000076531 14752210

[35] Schiessl H Frost HM Jee WS. Estrogen and bone-muscle strength and mass relationships. Bone (1998) 22:1–6. 10.1016/S8756-3282(97)00223-8 9437507

[36] Frost HM. An approach to estimating bone and joint loads and muscle strength in living subjects and skeletal remains. Am J Hum Biol (1999) 11:437–55. 10.1002/(SICI)1520-6300(1999)11:4<437::AID-AJHB4>3.3.CO;2-B 11533964

[37] Schoenau E. From mechanostat theory to development of the “functional muscle-bone-unit”. J Musculoskelet Neuronal Interact (2005) 5:232–8. 16172514

[38] Gussinyé M Terrades P Yeste D Vicens-Calvet E Carrascosa A. Low areal bone mineral density values in adolescents and young adult turner syndrome patients increase after long-term transdermal estradiol therapy. Horm Res (2000) 54:131–5. 10.1159/000053246 11357006

[39] Rao E Weiss B Fukami M Rump A Niesler B Mertz A Pseudoautosomal deletions encompassing a novel homeobox gene cause growth failure in idiopathic short stature and Turner syndrome. Nat Genet (1997) 16:54–63. 10.1038/ng0597-54 9140395

[40] Zinn AR Roeltgen D Stefanatos G Ramos P Elder FF Kushner H Turner syndrome neurocognitive phenotype maps to Xp22.3. Behav Brain Funct (2007) 3:24 10.1186/1744-9081-3-24 17517138 PMC1891305

[41] Vanderschueren D Venken K Ophoff J Bouillon R Boonen S Clinical review: sex steroids and the periosteum-reconsidering the roles of androgens and estrogens in periosteal expansion. J Clin Endocrinol Metab (2006) 91:378–82. 10.1210/jc.2005-1766 16303833

[42] Ross JL Quigley CA Cao D Feuillan P Kowal K Chipman JJ Growth hormone plus childhood low-dose estrogen in Turner’s syndrome. N Engl J Med (2011) 364:1230–42. 10.1056/NEJMoa1005669 21449786 PMC3083123

[43] Sylven L Hagenfeldt K Ringertz H Bone mineral density in middle-aged women with Turner’s syndrome. Eur J Endocrinol (1995) 132:47–52. 10.1530/eje.0.1320047 7850010

[44] Gravholt CH Naeraa RW Nyholm B Gerdes LU Christiansen E Schmitz O Glucose metabolism, lipid metabolism, and cardiovascular risk factors in adult Turner’s syndrome. The impact of sex hormone replacement. Diabetes Care (1998) 21:1062–70. 10.2337/diacare.21.7.1062 9653596

[45] Elsheikh M Bird R Casadei B Conway GS Wass JA The effect of hormone replacement therapy on cardiovascular hemodynamics in women with Turner’s syndrome. J Clin Endocrinol Metab (2000) 85:614–8. 10.1210/jcem.85.2.6384 10690864

[46] Trolle C Mortensen KH Hjerrild BE Cleemann L Gravholt CH Clinical care of adult Turner syndrome – new aspects. Pediatr Endocrinol Rev (2012) 9(Suppl 2):739–49. 22946288

[47] Sun L Peng Y Sharrow AC Iqbal J Zhang Z Papachristou DJ FSH directly regulates bone mass. Cell (2006) 125:247–60. 10.1016/j.cell.2006.01.051 16630814

[48] Iqbal J Sun L Kumar TR Blair HC Zaidi M. Follicle-stimulating hormone stimulates TNF production from immune cells to enhance osteoblast and osteoclast formation. Proc Natl Acad Sci U S A (2006) 103:14925–30. 10.1073/pnas.0606805103 17003115 PMC1595452

[49] Mori G D’Amelio P Faccio R Brunetti G. Bone-immune cell crosstalk: bone diseases. J Immunol Res (2015) 2015:108451. 10.1155/2015/108451 26000310 PMC4427089

[50] Colaianni G Cuscito C Colucci S. FSH and TSH in the regulation of bone mass: the pituitary/immune/bone axis. Clin Dev Immunol (2013) 2013:382698. 10.1155/2013/382698 23818914 PMC3683445

[51] Sowers MR Finkelstein JS Ettinger B Bondarenko I Neer RM Cauley JA The association of endogenous hormone concentrations and bone mineral density measures in pre- and perimenopausal women of four ethnic groups: SWAN. Osteoporos Int (2003) 14:44–52. 10.1007/s00198-002-1307-x 12577184

[52] Devleta B Adem B Senada S. Hypergonadotropic amenorrhea and bone density: new approach to an old problem. J Bone Miner Metab (2004) 22:360–4. 10.1007/s00774-004-0495-1 15221495

[53] Kawai H Furuhashi M Suganuma N. Serum follicle-stimulating hormone level is a predictor of bone mineral density in patients with hormone replacement therapy. Arch Gynecol Obstet (2004) 269:192–5. 10.1007/s00404-003-0532-7 13680264

[54] Hagen CP Main KM Kjaergaard S Juul A. FSH, LH, inhibin B and estradiol levels in Turner syndrome depend on age and karyotype: longitudinal study of 70 Turner girls with or without spontaneous puberty. Hum Reprod (2010) 25:3134–41. 10.1093/humrep/deq291 20956269

[55] Nakashima T Hayashi M Fukunaga T Kurata K Oh-Hora M Feng JQ Evidence for osteocyte regulation of bone homeostasis through RANKL expression. Nat Med (2011) 17:1231–4. 10.1038/nm.2452 21909105

[56] Cannon JG Kraj B Sloan G. Follicle-stimulating hormone promotes RANK expression on human monocytes. Cytokine (2011) 53:141–4. 10.1016/j.cyto.2010.11.011 21159522 PMC3021651

[57] Ellison JW Wardak Z Young MF Gehron Robey P Laig-Webster M Chiong W. PHOG, a candidate gene for involvement in the short stature of Turner syndrome. Hum Mol Genet (1997) 6:1341–7. 10.1093/hmg/6.8.1341 9259282

[58] Clement-Jones M Schiller S Rao E Blaschke RJ Zuniga A Zeller R The short stature homeobox gene SHOX is involved in skeletal abnormalities in Turner syndrome. Hum Mol Genet (2000) 9:695–702. 10.1093/hmg/9.5.695 10749976

[59] Carrel L Willard HF. X-inactivation profile reveals extensive variability in X-linked gene expression in females. Nature (2005) 434:400–4. 10.1038/nature03479 15772666

[60] Oliveira CS Alves C The role of the SHOX gene in the pathophysiology of Turner syndrome. Endocrinol Nutr (2011) 58:433–42. 10.1016/j.endonu.2011.06.005 21925981

[61] Decker E Durand C Bender S Rödelsperger C Glaser A Hecht J FGFR3 is a target of the homeobox transcription factor SHOX in limb development. Hum Mol Genet (2011) 20:1524–35. 10.1093/hmg/ddr030 21273290

[62] Aza-Carmona M Shears DJ Yuste-Checa P Barca-Tierno V Hisado-Oliva A Belinchón A SHOX interacts with the chondrogenic transcription factors SOX5 and SOX6 to activate the aggrecan enhancer. Hum Mol Genet (2011) 20:1547–59. 10.1093/hmg/ddr032 21262861

[63] Frederiksen AL Hansen S Brixen K Frost M. Increased cortical area and thickness in the distal radius in subjects with SHOX-gene mutation. Bone (2014) 69:23–9. 10.1016/j.bone.2014.09.001 25220427

[64] Rajpathak SN Vellarikkal SK Patowary A Scaria V Sivasubbu S Deobagkar DD. Human 45,X fibroblast transcriptome reveals distinct differentially expressed genes including long noncoding RNAs potentially associated with the pathophysiology of Turner syndrome. PLoS One (2014) 9:e100076. 10.1371/journal.pone.0100076 24932682 PMC4059722

[65] McCoy RJ Widaa A Watters KM Wuerstle M Stallings RL Duffy GP Orchestrating osteogenic differentiation of mesenchymal stem cells-identification of placental growth factor as a mechanosensitive gene with a pro-osteogenic role. Stem Cells (2013) 31:2420–31. 10.1002/stem.1482 23897668

[66] Maes C Coenegrachts L Stockmans I Daci E Luttun A Petryk A Placental growth factor mediates mesenchymal cell development, cartilage turnover, and bone remodeling during fracture repair. J Clin Invest (2006) 116:1230–42. 10.1172/JCI26772 16614757 PMC1435721

[67] Marrony S Bassilana F Seuwen K Keller H. Bone morphogenetic protein 2 induces placental growth factor in mesenchymal stem cells. Bone (2003) 33:426–33. 10.1016/S8756-3282(03)00195-9 13678785

[68] Gerstenfeld LC Einhorn TA. COX inhibitors and their effects on bone healing. Expert Opin Drug Saf (2004) 3:131–6. 10.1517/14740338.3.2.131 15006719

[69] Myers LK Bhattacharya SD Herring PA Xing Z Goorha S Smith RA The isozyme-specific effects of cyclooxygenase-deficiency on bone in mice. Bone (2006) 39:1048–52. 10.1016/j.bone.2006.05.015 16875891

[70] Chen L Jiang W Huang J He BC Zuo GW Zhang W Insulin-like growth factor 2 (IGF-2) potentiates BMP-9-induced osteogenic differentiation and bone formation. J Bone Miner Res (2010) 25:2447–59. 10.1002/jbmr.133 20499340 PMC3179288

[71] Bodine PV Zhao W Kharode YP Bex FJ Lambert AJ Goad MB The Wnt antagonist secreted frizzled-related protein-1 is a negative regulator of trabecular bone formation in adult mice. Mol Endocrinol (2004) 18:1222–37. 10.1210/me.2003-0498 14976225

[72] Gaur T Rich L Lengner CJ Hussain S Trevant B Ayers D Secreted frizzled related protein 1 regulates Wnt signaling for BMP2 induced chondrocyte differentiation. J Cell Physiol (2006) 208:87–96. 10.1002/jcp.20637 16575902

[73] Neely EK Marcus R Rosenfeld RG Bachrach LK. Turner syndrome adolescents receiving growth hormone are not osteopenic. J Clin Endocrinol Metab (1993) 76:861–6. 10.1210/jcem.76.4.8473397 8473397

[74] Gravholt CH Hjerrild BE Mosekilde L Hansen TK Rasmussen LM Frystyk J Body composition is distinctly altered in Turner syndrome: relations to glucose metabolism, circulating adipokines, and endothelial adhesion molecules. Eur J Endocrinol (2006) 155:583–92. 10.1530/eje.1.02267 16990658

[75] Elsheikh M Conway GS. The impact of obesity on cardiovascular risk factors in Turner’s syndrome. Clin Endocrinol (1998) 49:447–50. 10.1046/j.1365-2265.1998.00552.x 9876341

[76] Bakalov VK Cooley MM Quon MJ Luo ML Yanovski JA Nelson LM Impaired insulin secretion in the Turner metabolic syndrome. J Clin Endocrinol Metab (2004) 89:3516–20. 10.1210/jc.2004-0122 15240640

[77] Cao JJ. Effects of obesity on bone metabolism. J Orthop Surg Res (2011) 6:30. 10.1186/1749-799X-6-30 21676245 PMC3141563

[78] Gimble JM Robinson CE Wu X Kelly KA Rodriguez BR Kliewer SA Peroxisome proliferator-activated receptor-gamma activation by thiazolidinediones induces adipogenesis in bone marrow stromal cells. Mol Pharmacol (1996) 50:1087–94. 8913339

[79] Sen B Xie Z Case N Ma M Rubin C Rubin J Mechanical strain inhibits adipogenesis in mesenchymal stem cells by stimulating a durable betacatenin signal. Endocrinology (2008) 149:6065–75. 10.1210/en.2008-0687 18687779 PMC2613068

[80] Wellen KE Hotamisligil GS. Obesity-induced inflammatory changes in adipose tissue. J Clin Invest (2003) 112:1785–8. 10.1172/JCI20514 14679172 PMC297006

[81] Mundy GR. Osteoporosis and inflammation. Nutr Rev (2007) 65:S147–51. 10.1111/j.1753-4887.2007.tb00353.x 18240539

[82] Leidig-Bruckner G Grobholz S Bruckner T Scheidt-Nave C Nawroth P Schneider JG. Prevalence and determinants of osteoporosis in patients with type 1 and type 2 diabetes mellitus. BMC Endocr Disord (2014) 14:33. 10.1186/1472-6823-14-33 24721668 PMC4021186

[83] Jackuliak P Payer J. Osteoporosis, fractures, and diabetes. Int J Endocrinol (2014) 2014:820615. 10.1155/2014/820615 25050121 PMC4094869

[84] Krela-Kaźmierczak I Szymczak A Łykowska-Szuber L Eder P Linke K. Osteoporosis in gastrointestinal diseases. Adv Clin Exp Med (2016) 25:185–90. 10.17219/acem/33746 26935513

[85] Castelo-Branco C. Management of Turner syndrome in adult life and beyond. Maturitas (2014) 79:471–5. 10.1016/j.maturitas.2014.08.011 25438673

[86] Tuchendler D Bolanowski M. The influence of thyroid dysfunction on bone metabolism. Thyroid Res (2014) 7:12. 10.1186/s13044-014-0012-0 25648501 PMC4314789

[87] Rubin K. Transitioning the patient with Turner’s syndrome from pediatric to adult care. J Pediatr Endocrinol Metab (2003) 16(Suppl 3):651–9. 12795368

[88] Collett-Solberg PF Gallicchio CT Coelho SC Siqueira RA Alves ST Guimarães MM. Endocrine diseases, perspectives and care in Turner syndrome. Arq Bras Endocrinol Metabol (2011) 55:550–8. 10.1590/S0004-27302011000800008 22218436

[89] Nadeem M Roche EF. Bone health in children and adolescent with Turner syndrome. J Pediatr Endocrinol Metab (2012) 25:823–33. 10.1515/jpem-2012-0088 23426807

[90] Naeraa RW Brixen K Hansen RM Hasling C Mosekilde L Andresen JH Skeletal size and bone mineral content in Turner’s syndrome: relation to karyotype, estrogen treatment, physical fitness, and bone turnover. Calcif Tissue Int (1991) 49:77–83. 10.1007/BF02565125 1913298

[91] Cleemann L Hjerrild BE Lauridsen AL Heickendorff L Christiansen JS Mosekilde L Long-term hormone replacement therapy preserves bone mineral density in Turner syndrome. Eur J Endocrinol (2009) 161:251–7. 10.1530/EJE-09-0020 19447901

